# Ultra-sensitive Nanoprobe Modified with Tumor Cell Membrane for UCL/MRI/PET Multimodality Precise Imaging of Triple-Negative Breast Cancer

**DOI:** 10.1007/s40820-020-0396-4

**Published:** 2020-02-22

**Authors:** Hanyi Fang, Mengting Li, Qingyao Liu, Yongkang Gai, Lujie Yuan, Sheng Wang, Xiao Zhang, Min Ye, Yongxue Zhang, Mingyuan Gao, Yi Hou, Xiaoli Lan

**Affiliations:** 1 Department of Nuclear Medicine, Union Hospital, Tongji Medical College, Huazhong University of Science and Technology, Wuhan, 430022 People’s Republic of China; 2 Hubei Province Key Laboratory of Molecular Imaging, Wuhan, 430022 People’s Republic of China; 3 School of Pharmacy, Tongji Medical College, Huazhong University of Science and Technology, Wuhan, 430030 People’s Republic of China; 4 Center for Molecular Imaging and Nuclear Medicine, State Key Laboratory of Radiation Medicine and Protection, School for Radiological and Interdisciplinary Sciences (RAD-X), Soochow University, Suzhou, 215123 Jiangsu People’s Republic of China; 5 Key Laboratory of Colloid, Interface and Chemical Thermodynamics, Institute of Chemistry, Chinese Academy of Sciences, Beijing, 100190 People’s Republic of China

**Keywords:** Triple-negative breast cancer, Molecular classification, Multimodality imaging, Cancer cell membranes, Upconversion

## Abstract

Triple-negative breast cancer (TNBC) is a subtype of breast cancer in which the estrogen receptor and progesterone receptor are not expressed, and human epidermal growth factor receptor 2 is not amplified or overexpressed either, which make the clinical diagnosis and treatment very challenging. Molecular imaging can provide an effective way to diagnose TNBC. Upconversion nanoparticles (UCNPs), are a promising new generation of molecular imaging probes. However, UCNPs still need to be improved for tumor-targeting ability and biocompatibility. This study describes a novel probe based on cancer cell membrane-coated upconversion nanoparticles (CCm-UCNPs), owing to the low immunogenicity and homologous-targeting ability of cancer cell membranes, and modified multifunctional UCNPs. This probe exhibits excellent performance in breast cancer molecular classification and TNBC diagnosis through UCL/MRI/PET tri-modality imaging in vivo. By using this probe, MDA-MB-231 was successfully differentiated between MCF-7 tumor models in vivo. Based on the tumor imaging and molecular classification results, the probe is also expected to be modified for drug delivery in the future, contributing to the treatment of TNBC. The combination of nanoparticles with biomimetic cell membranes has the potential for multiple clinical applications.

**Electronic supplementary material:**

The online version of this article (10.1007/s40820-020-0396-4) contains supplementary material, which is available to authorized users.

## Highlights

- A biomimetic nanoprobe was built with cancer cell membrane-coated and Gd^3+^-doped upconversion nanoparticles.
- The nanoprobe could be applied to in vivo UCL/MRI/PET multimodality precise imaging and successfully differentiated MDA-MB-231 tumor models through in vivo tri-modality imaging, which may be used for breast cancer molecular classification.

**Electronic supplementary material:**

The online version of this article (10.1007/s40820-020-0396-4) contains supplementary material, which is available to authorized users.

## Graphical abstract

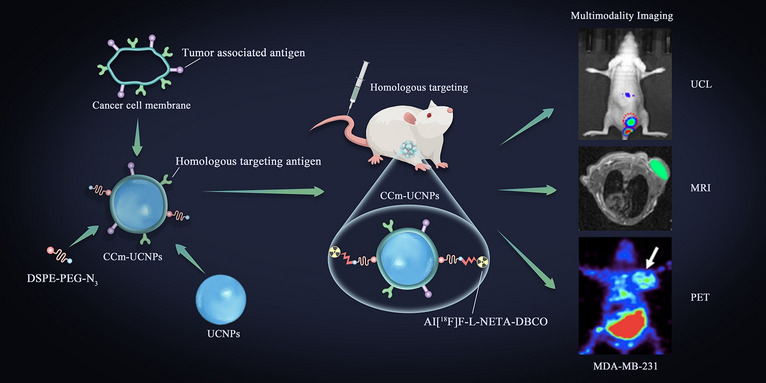

## Introduction

Breast cancer (BC) is a malignant disease leading to approximately two million new cases (11.6%) and 620,000 deaths worldwide in 2018 [1]. It is also a highly heterogeneous disease requiring molecular classification for treatment and prognosis [2]. Triple-negative breast cancer (TNBC) is a subtype of BC in which the estrogen receptor (ER) and progesterone receptor (PR) are not expressed, and the human epidermal growth factor receptor 2 (HER2) is not amplified or overexpressed either [3]. At present, the diagnosis of TNBC mostly relies on tissue biopsy, which is affected by sampling error and invasiveness [4]. Therefore, the clinical diagnosis of TNBC remains challenging.

Molecular imaging can in principle provide powerful tools for identifying cancers with greatly improved specificity and sensitivity [5], and functional inorganic nanoparticles have shown great potential as imaging probes owing to the intrinsic physical properties of inorganic particles apart from the well-known enhanced permeation and retention effect of tumors for nano-objects. Among different kinds of functional inorganic nanoparticles, upconversion nanoparticles (UCNPs) are superior as light-emitting, and rare-earth ions can facilely be combined with paramagnetic ones such as Gd^3+^, for simultaneously visualizing tumors through upconversion luminescence (UCL) [6] and magnetic resonance imaging (MRI) [7, 8]. Besides, they also provide an excellent platform to further combine photoacoustic imaging (PAI) [9], single-photon emission computed tomography (SPECT) [10], and positron emission tomography (PET) imaging [11], with above imaging modalities.

Every imaging modality has its intrinsic advantages and disadvantages. To acquire multi-dimensional biological information, rationally combining different imaging modalities is essentially required, thus receiving increasing attention [7, 11–13]. The UCL of rare-earth nanoparticles typically requires near-infrared (NIR) lasers of 800 or 980 nm as excitation source which can reach deeper tissues and avoid autofluorescence of the biological tissues [14], particularly suitable for BC detection. The excellent photostability, lack of photoblinking, and ultra-sensitive detection of upconverting visible emissions make UCNPs promising for bioimaging [14], even for real-time imaging [15, 16]. MRI can provide high spatial resolution of soft tissues [17]. PET exhibits high sensitivity and unlimited detection depth [13]. UCNPs can well combine these strengths by being properly incorporated with Gd^3+^ and SPECT/PET nuclides [7, 11, 12]. However, the nanoparticles are prone to stimulate the mononuclear phagocyte system, shortening their blood residence time [18]. Therefore, different types of surface engineering approaches have been developed by modifying the nanoparticles with either artificial materials or natural substances. Poly(ethylene glycol) (PEG) [19] and cell membranes [15, 20, 21] are two representative examples of the aforementioned approaches. Recently, cell membranes such as the erythrocyte membrane [22–24], platelet membrane [25–27], and cancer cell membrane [16], have been gaining increasing attention. In comparison with polymer coating, cell membranes present low immunogenicity and may provide homologous-targeting ability if cancer cell membrane (CCm) is adopted [12, 16, 28].

**Scheme 1:**
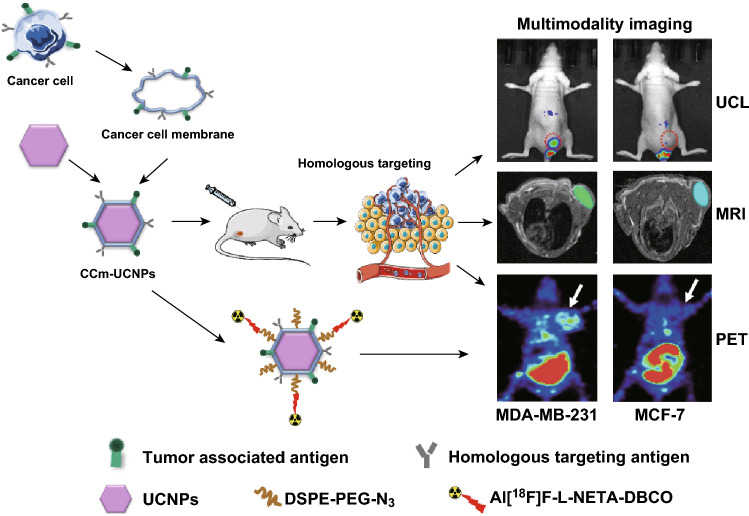
Illustration of the cancer cell membrane-coated Gd^3+^-doped upconversion nanoparticles (CCm-UCNPs) used for differentiating between MDA-MB-231 and MCF-7 tumor-bearing mice models by homologous-targeting multimodality imaging, including UCL, MRI, and PET. None of the mice shown in figure was the same mouse

In this study, we used the cancer cell membrane of MDA-MB-231, a kind of TNBC cell, to modify Gd^3+^-doped upconversion nanoparticles NaGdF_4_:Yb,Tm@NaGdF_4_ (CCm_231_-UCNPs) for in vivo UCL/MRI/PET tri-modality tumor imaging of BC and further differentiate between breast cancer subtypes of MDA-MB-231 and MCF-7 to demonstrate the potential of CCm-UCNPs in BC molecular classification (Scheme 1).

## Materials and Methods

### Materials

UCNPs (NaGdF_4_:Yb,Tm@NaGdF_4_) were supplied by Gao’s research group [10]. Dulbecco’s modified Eagle medium (DMEM), RPMI-1640 medium, phosphate buffer saline (PBS), trypsin and ethylenediaminetetraacetic acid (EDTA), and penicillin–streptomycin were purchased from Gibco Life Technologies (Gaithersburg, MD, USA). Fetal bovine serum (FBS) was purchased from ScienCell (Carlsbad, CA, USA). Leibovitz’s L15 medium, 4′,6-diamidino-2-phenylindole (DAPI), paraformaldehyde, and Cell Counting Kit-8 (CCK-8) were purchased from Boster Biotechnology (Wuhan, China). 1, 2-Distearoyl-sn-glycero-3-phosphoethanolamine-N-[Cy5(polyethylene glycol)-2000] (DSPE-PEG-Cy5), dibenzocyclooctyne (DBCO), and 1, 2-distearoyl-sn-glycero-3-phosphoethanolamine-N-[azido (polyethylene glycol)-2000] (DSPE-PEG-N_3_) were purchased from Sigma-Aldrich (St. Louis, MO, USA). All of the aqueous solutions were prepared using deionized (DI) water purified with a purification system. The other reagents used in this work were purchased from Aladdin-Reagent (Shanghai, China).

### Preparation of Cancer Cell Membranes

Cancer cell membrane-derived vesicles (CCm) were prepared according to the previous report [12, 16, 29]. MDA-MB-231 human breast cancer cells were maintained in DMEM supplemented with 10% FBS and 1% penicillin–streptomycin. To harvest membranes, cancer cells were grown in T-175 culture flasks to full confluence and detached with 2 mM EDTA in PBS and washed in PBS three times by centrifuging at 1000 rpm for 4 min. The cells were suspended in a hypotonic lysing buffer consisting of 20 mM Tris-HCl, 10 mM KCl, 2 mM MgCl_2_, and 1 EDTA-free mini protease inhibitor tablet per 10 mL of solution and disrupted at 4 °C overnight. The entire solution was ultrasonically dispersed by an ultrasonic homogenizer (Scientz-IID, Ningbo Scientz Biotechnology Co., Ltd., Ningbo, China), before spinning down at 3200 g for 5 min. The supernatants were saved while the pellet was resuspended in hypotonic lysing buffer and dispersed by ultrasound again. The supernatants were pooled and centrifuged at 20,000 g for 20 min, after which the pellet was discarded and the supernatant was centrifuged again at 100,000 g for 1 h using an ultra-speed centrifuge (Optima XPN-100 Ultracentrifuge, Beckman Coulter, Miami, FL, USA). The pellet containing the plasma membrane material was then washed once with 10 mM Tris-HCl and 1 mM EDTA. The final pellet was collected and used as purified cancer cell membranes for subsequent experiments.

### Characterization of Cancer Cell Membrane Protein

SDS-PAGE gel electrophoresis displayed for protein characterization. All samples were prepared at a final protein concentration of 1 mg mL^−1^ in loading buffer as measured by a bicinchoninic acid (BCA) assay. CCm_231_-UCNPs were purified by centrifugation at 13,000 rpm to pellet the coated particles but not free vesicles or protein. The mixture of samples and loading buffer with the volume ratio of 4:1 was heated to 100 °C for 10 min, and the mixture of samples and loading buffer with the volume ratio of 3:1 was heated to 37 °C for 30 min. The denatured sample was loaded into each well in an Electrophoresis System (Cat #DYZC-24DN, Beijing Liuyi Biotechnology Co., Ltd., Beijing, China) based on the manufacturer’s instructions. Protein staining was accomplished using 0.05% Coomassie Brilliant Blue R-250, 30% methanol, and 10% acetic acid for 3 h and destained in 30% methanol and 10% acetic acid and stored at room temperature. For western blot analysis, the protein was transferred to polyvinylidene fluoride (PVDF) membranes (Millipore Cat#IPVH00010, Millipore, Inc. Bedford, MD, USA) using an XCell II Blot Module (Invitrogen, Carlsbad, CA, USA) in NuPAGE transfer buffer (Invitrogen, Carlsbad, CA, USA) per manufacturer’s instructions. Membranes were probed using antibodies against EGFR, Na^+^/K^+^-ATPase, histone H3, and glyceraldehyde 3-phosphate dehydrogenase along with either horseradish peroxidase (HRP)-conjugated anti-mouse IgG (Poly4053, Biolegend, San Diego, CA, USA) or anti-rabbit IgG (Poly4064, Biolegend). Films were developed using ECL western blotting substrate (Pierce Biotechnology, Rockford, IL, USA) and developed with the Mini-Medical/90 Developer (ImageWorks, Culver City, CA, USA).

### In Vitro and Cell Cytotoxicity Assay

1, 2-Distearoyl-sn-glycero-3-phosphoethanolamine-N-(polyethylene glycol) 2000-Cy5 (DSPE-PEG-Cy5) was cubed with CCm_231_ for 30 min at 37 °C to obtain Cy5-DSPE-PEG-CCm_231_ and mixing them with UCNPs. The mixture was subsequently extruded 11 times through 400 nm polycarbonate membrane to get Cy5-DSPE-PEG-CCm_231_-UCNPs. Cells were incubated 4 h with Cy5-DSPE-PEG-CCm_231_-UCNPs. To exclude the effect of DSPE-PEG-Cy5, MDA-MB-231 cells were incubated with DSPE-PEG-Cy5 for 4 h. Cell nuclei were stained with 4′,6-diamidino-2-phenylindole (DAPI), and confocal laser scanning microscopy (CLSM) under an external 650 nm laser was used to image.

A CCK-8 assay was used to evaluate the cytotoxicity of nanoparticles to MDA-MB-231 cancer cells. Cells were seeded in 96-well plates at a density of 3 × 10^3^ cells per well and cultured for 12 h. Then UCNPs and CCm_231_-UCNPs at various concentrations (i.e., 5, 25, 50, 100, and 500 μg mL^−1^) were added to the medium, and the cells were incubated for another 24 h. The cells grown without any nanoparticles were used as a control. At the end of the incubation, 5 mg mL^−1^ CCK-8 PBS solution was added, and the plate was incubated for another 4 h. Finally, the absorbance values of the cells per well were determined with a microplate reader (Bio-Rad, Hercules, CA, USA) at 450 nm for analyzing the cell viability. The background absorbance of the well plate was measured and subtracted. The cytotoxicity was calculated by dividing the optical density (OD) values of treated groups (*T*) by the OD values of the control (*C*) (*T*/*C* × 100%).

### Animals and Tumor Model

Animals received care under the instruction of the Guidance Suggestions for the Care and Use of Laboratory Animals. Four- to six-week-old female BALB/c nude mice (Beijing HuaFuKang Bioscience Co. Ltd, China) were subcutaneously injected with 100 μL serum-free PBS containing 5 × 10^6^ MDA-MB-231 cells or MCF-7 cells into the upper limbs or lower limbs of each mouse, according to the imaging situation. About 1 week after the injection, when the tumor volume reached 60–250 mm^3^, the tumor-bearing mice model would be used for further experiments.

### In Vivo Imaging

When the volumes of MDA-MB-231 tumor or MCF-7 tumor reached 60–250 mm^3^, the BALB/c nude mice were divided into groups randomly and received an i.v. injection of 200 μL PBS or PBS containing the different nanoparticle preparations (i.e., UCNPs, RBC-UCNPs, and CCm_231_-UCNPs) at the concentration of 5 mg mL^−1^ via the tail vein. All mice were anesthetized by isoflurane. For in vivo UCL imaging, the fluorescence signals were obtained by an ex/in vivo imaging system (IVIS Lumina XRMS Series III, PerkinElmer Inc.) equipped with fluorescent filter sets (excitation/emission = 980/790 nm) at 0, 3, 6, 12, 24, 36, and 48 h after the injection. Then all mice were killed to obtain the tumor and major organs (heart, liver, spleen, lung, kidneys) to conduct the ex vivo UCL signals through the ex/in vivo imaging system (IVIS Lumina XRMS Series III, PerkinElmer Inc.).

For in vivo MR imaging, the dose level was set to 15 mg of Gd per kilogram body weight for CCm_231_-UCNPs, RBCm-UCNPs, UCNPs, and Gd-DTPA. The MR images were acquired on a 7.0 T animal MRI instrument (BioSpec 70/20 USR, Bruker, Karlsruhe, Germany) after 24-h injection of CCm_231_-UCNPs, RBCm-UCNPs, and UCNPs. As for the Gd-DTPA group, images were acquired immediately after the injection of Gd-DTPA. The detailed imaging parameters were set as follows: echo time (TE) = 15.3 ms; repetition time (TR) = 500, 1000, 1500, 2000 ms; number of excitations (NEX) = 8 [8].

PET imaging was performed on a micro-PET (Trans-PET^®^ BioCaliburn^®^ LH, Raycan Technology Co., Ltd., Suzhou, China). 1, 2-Distearoyl-sn-glycero-3-phosphoethanolamine-N-[azido (polyethylene glycol)-2000] (DSPE-PEG-N_3_) was incubated with CCm_231_ for 30 min at 37 °C to form N_3_-PEG-DSPE-CCm_231_-UCNPs. The tumor-bearing mice were first injected with N_3_-DSPE-PEG-CCm_231_-UCNPs, N_3_-DSPE-PEG-RBCm-UCNPs, or N_3_-DSPE-PEG, and then 24 h after the injection, Al[^18^F]F-L-NETA-DBCO was subsequently injected into the tumor-bearing mice via tail vein. First, L-NETA-DBCO was successfully radiolabeled with ^18^F via Al-^18^F chelation [33], and then, ^18^F-labeled aza-dibenzocyclooctyne (DBCO) radioligands (Al[^18^F]F-L-NETA-DBCO) were conjugated with azide-modified UCNPs by in vivo strain-promoted alkyne azide cycloaddition (SPAAC), which enables PET imaging [34]. PET static imaging was performed at 0.5, 1, 2, and 4 h after the injection of Al[^18^F]F-L-NETA-DBCO.

To measure the stability of the probe Al[^18^F]F-L-NETA-DBCO, quality control was performed by high-performance liquid chromatography (HPLC) at 0, 0.5, 1, 2, and 4 h. In vivo stability testing was also conducted. After injection of the probe Al[^18^F]F-L-NETA-DBCO into BALB/c nude mice, the blood was obtained at 2 h and 4 h after the injection and analyzed with HPLC.

### Biodistribution Studies

MDA-MB-231 or MCF-7 tumor-bearing mice injected with N_3_-DSPE-PEG-CCm_231_-UCNPs and Al[^18^F]F-L-NETA-DBCO to evaluate whole-body elimination and excretion were killed by cervical dislocation under anesthesia using isoflurane; samples of blood, normal tissues (including brain, heart, lung, liver, spleen, kidney, stomach, small intestine, large intestine, muscle, bone), and tumor were collected and weighed; and the radioactivity in each was measured in a γ-counter. Tissue radioactivity was calculated as percent injected dose/g (% ID/g) and then converted to % ID/organ using previously determined standard organ weights. The concentration of Gd^3+^ in each organ was then measured by inductively coupled plasma-atomic emission spectroscopy (ICP-AES). For this analysis, samples were prepared as described by Rao et al. [16]. ICP-AES was performed using a Prodigy 7 (Leeman Labs Inc., Hudson, NH, USA) instrument, and the concentration (mg L^−1^) of Gd^3+^ in the tissues was obtained by reference to a standard curve.

### Biotoxicity Evaluation

For evaluating systematic toxicity, BALB/c nude mice (*n* = 6) received an injection of 200 μL of PBS, or PBS containing UCNPs or CCm_231_-UCNPs at a concentration of 5 mg mL^−1^, or PBS containing equivalent numbers of CCm_231_-vesicles. Death and body weight were observed for 30 days. On the 30th day after the injection, all the mice were euthanized and their blood and major organs were collected for blood biochemistry (red blood cells (RBCs), white blood cells (WBCs), platelets (PLT), hemoglobin (HGB), hematocrit (HCT), mean corpuscular volume (MCV), mean corpuscular hemoglobin (MCH) and mean corpuscular hemoglobin concentration (MCHC)), hematology tests (alkaline phosphatase (ALP), aspartate aminotransferase (AST), alanine transaminase (ALT), creatinine (CRE) and blood urea nitrogen (BUN)), and histology analysis (hematoxylin and eosin (H&E)-stained slices).

### Statistical Analysis

Results are expressed as mean ± standard error of the mean. Data analyses were conducted using the software GraphPad Prism 6.0 (GraphPad Software, San Diego, CA, USA). The differences among groups were analyzed using one-way ANOVA followed by Tukey’s post-test. *P* value of < 0.05 indicates statistical significance.

## Results and Discussion

### Preparation and Characterization of CCm-UCNPs

**Fig. 1:**
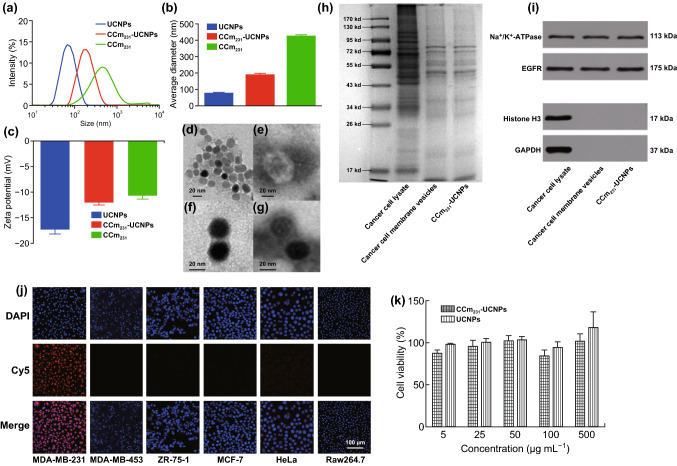
Physicochemical characterization of CCm_231_-UCNPs. **a** Size intensity curves, **b** hydrodynamic size, and **c** zeta potential of UCNPs, CCm_231_-UCNPs, and CCm_231_ measured by dynamic light scattering (DLS). TEM images of **d** UCNPs, **e** CCm_231_, and **f**, **g** CCm_231_-UCNPs. The TEM samples were negatively stained with phosphotungstic acid. The scale bars = 20 nm. **h** SDS-PAGE protein analysis of MDA-MB-231 cancer cell lysate, cancer cell membrane vesicles, and CCm_231_-UCNPs. Samples were run at equal protein concentration and stained with Coomassie Blue. **i** Western blotting analysis. Samples were run at equal protein concentration and immunostained against membrane markers including Na^+^/K^+^-ATPase, EGFR, histone H3 (a nuclear marker), and glyceraldehyde 3-phosphate dehydrogenase (a cytosolic marker). In vitro homologous-targeting ability and cytotoxicity of CCm_231_-UCNPs. **j** Confocal laser scanning microscopy (CLSM) images of various cells after incubation with CCm_231_-UCNPs. The scale bars = 100 μm. **k** Survival of MDA-MB-231 cells treated with different concentrations of CCm_231_-UCNPs and UCNPs. The data points represent mean ± SD (*n* = 3)

To construct CCm-UCNPs, MDA-MB-231 cells, as the membrane source, were processed by using the method reported by Rao et al. [16]. Briefly, the membrane derivation was achieved through a combination of hypotonic lysis, mechanical membrane disruption, and differential centrifugation. With the collected membranes, CCm vesicles were then formed by physical extrusion through a 400-nm porous polycarbonate membrane on a mini extruder. Then, mixing UCNPs with the membranes by ultrasound and repeating the physical extrusion through a 200-nm pore, CCm was then coated by physical extrusion to form CCm_231_-UCNPs. As measured by dynamic light scattering (DLS), the hydrodynamic size of the UCNPs was approximate 75 nm (Fig. 1a, b), while that of CCm_231_ was around 400 nm. Upon optimization of UCNPs:CCm_231_ ratio, CCm_231_-UCNPs of approximately 200 nm were obtained, which presented the best hydrodynamic size and polydispersity in comparison with those obtained with different UCNPs:CCm_231_ ratios (Fig. S1a). The zeta-potential measurements shown in Fig. 1c exhibited that the surface potential of CCm_231_-UCNPs was much closer to CCm_231_ rather than that of the mother UCNPs, indicating that CCm_231_ coating was successfully achieved. Transmission electron microscopy (TEM) is shown in Fig. 1d–g, the membrane coating around the UCNPs can be visualized with a thickness of around 3 nm, and the CCm_231_ evenly was coated around the UNCP cores of ~ 25 nm. The as-prepared CCm_231_-UCNPs presented good colloidal stability in 1 × phosphate-buffered saline (PBS) for 3 days (Fig. S1b). Also, the upconversion luminescence CCm_231_-UCNPs remained nearly unaltered in comparison with that of UCNPs under excitation at 980 nm (Fig. S1c), indicating that CCm coating did not affect the upconversion luminescence of UCNPs.

To confirm the existence of the CCm proteins on the UCNPs, the CCm_231_-UCNPs were subjected to sodium dodecyl sulfate–polyacrylamide gel electrophoresis (SDS–PAGE) and western blotting analysis. The SDS–PAGE results shown in Fig. 1h revealed that CCm_231_-UCNPs presented a protein profile very similar to those of CCm_231_ and MDA-MB-231 cell lysate. The western blotting analysis confirmed that the CCm_231_-UCNPs well inherited the membrane-specific markers such as Na^+^/K^+^-ATPase and positive antigen EGFR from the MDA-MB-231 cells, while the control nuclear protein marker (i.e., histone H3) and the cytosol marker (i.e., glyceraldehyde 3-phosphate dehydrogenase (GAPDH)) were almost undetectable from the final CCm_231_-UCNPs, as shown in Fig. 1i.

### In Vitro Homologous Targeting and Cytotoxicity of CCm_231_-UCNPs

It was expected that CCm_231_ coating would endow the CCm_231_-UCNPs with homologous-targeting ability. To evaluate such ability of CCm_231_-UCNPs in targeting MDA-MB-231 cells, 1, 2-distearoyl-sn-glycero-3-phosphoethanolamine-N-(polyethylene glycol) 2000-Cy5 (DSPE-PEG-Cy5), a kind of cyanine dye, was inserted into CCm_231_ lipid bilayer to achieve Cy5-labeled CCm_231_-UCNPs [32], denoted as Cy5-DSPE-PEG-CCm_231_-UCNPs. Then, different types of tumor cells including human breast cancer cells MDA-MB-231, MCF-7, ZR-75-1, MDA-MB-453 (the phenotypes of various cells are shown in Table S1), and human cervical cancer cells (HeLa) were adopted to show the binding affinity of Cy5-DSPE-PEG-CCm_231_-UCNPs after co-incubated with the corresponding cells. The confocal laser scanning microscopy (CLSM) results given in Fig. 1j revealed that only MDA-MB-231 cells presented the strongest fluorescence after incubation with Cy5-DSPE-PEG-CCm_231_-UCNPs. These confirmed the specific homologous-targeting ability of CCm_231_-UCNPs to MDA-MB-231 cells. There is some evidence proving that cancer cell membranes play an important role in immune tolerance in the tumor microenvironment [29]. To investigate the low immunogenicity of CCm_231_-UCNPs, murine macrophage-like cells (Raw264.7) were used. The Raw264.7 cells had nearly no uptake of Cy5-DSPE-PEG-CCm_231_-UCNPs (Fig. 1j), indicating that CCm-UCNPs can obscure their identification by the mononuclear phagocyte system and indeed decreased immunogenicity. And MDA-MB-231 cells showed no uptake of DSPE-PEG-Cy5, which could exclude the effect of DSPE-PEG-Cy5 (Fig. S2).

The cholecystokinin (CCK)-8 assay was used to evaluate the cytotoxicity of UCNPs and CCm_231_-UCNPs to MDA-MB-231 cells. MDA-MB-231 cells were co-incubated with UCNPs and CCm_231_-UCNPs at concentrations up to 500 μg mL^−1^ for 24 h. The results revealed that the survival rates of cells in the UCNPs group and the CCm_231_-UCNPs group were both > 80% (Fig. 1k), indicating UCNPs and CCm_231_-UCNPs exerted no obvious toxicity toward MDA-MB-231 cells.

### In Vivo UCL/MRI/PET Imaging

In this work, we speculated that CCm_231_-UCNPs would exhibit the homologous-targeting ability to MDA-MB-231 cancer cells in vivo. We set red blood cell membrane-coated UCNPs (RBCm-UCNPs) in PBS as a control to compare the results of homologous targeting and passive targeting. PBS or PBS containing CCm_231_-UCNPs, RBCm-UCNPs, or UCNPs at the same concentration were injected into MDA-MB-231 subcutaneous-tumor-bearing BALB/c nude mice through the tail vein. To avoid the impact of liver uptake on tumor area imaging, we injected cancer cells into the lower limb of the mice models [12, 15, 16, 29].

**Fig. 2:**
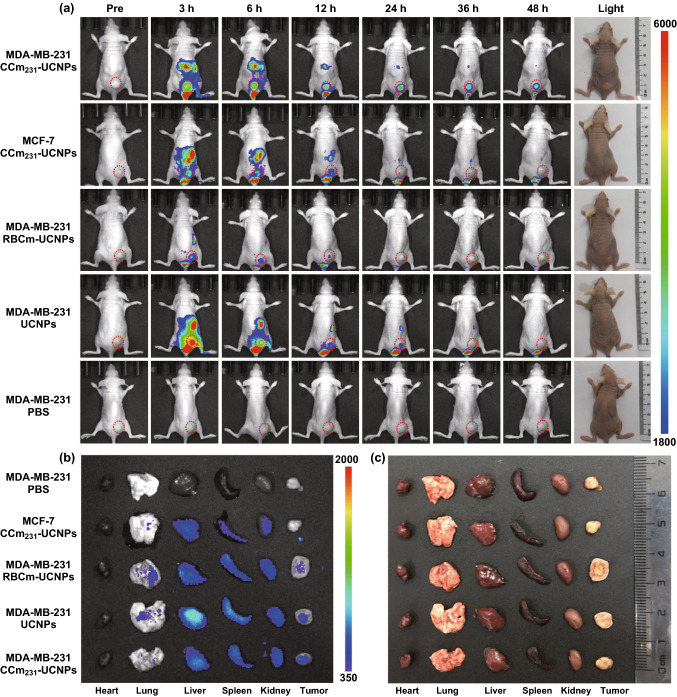
Homologous-targeting evaluation and in vivo upconversion luminescence (UCL) imaging. **a** BALB/c nude tumor-bearing mice injected with PBS or PBS containing UCNPs, RBCm-UCNPs, and CCm_231_-UCNPs at different times after the injection. **b**, **c** Ex vivo UCL images of tumors and major organs of the killed mice at 48 h after the injection

For in vivo UCL imaging, the fluorescence signals were obtained after the injection. As shown in Fig. 2a, the CCm_231_-UCNPs group displayed the strongest UCL signal in the tumor site, demonstrating the homologous binding ability of CCm_231_-UCNPs to target MDA-MB-231 tumors. The RBCm-UCNPs group showed similar tumor accumulation to the UCNPs group attributed to the EPR effect, but lower fluorescence signal compared to that of the CCm_231_-UCNPs group. Liver is one of the primary organs of the phagocyte-enriched reticuloendothelial system (RES) [30] and can accumulate the most nanoparticles [31]. Liver accumulation by the CCm_231_-UCNPs group was much lower than that of the UCNPs group, indicating that CCm coating can indeed decrease the RES uptake. In the CCm_231_-UCNPs group, the liver signal was of short duration, almost disappeared in 24 h, while the tumor signal lasted much longer, up to 48 h. The best imaging time was at 24 h after the injection with high uptake by the tumor and low uptake by the liver, indicating the feasibility of using the CCm_231_-UCNPs for imaging and treatment delivery. To investigate the homologous-targeting ability, CCm_231_-UCNPs were injected into MDA-MB-231 and MCF-7 tumor-bearing mice. The UCL signal in the MDA-MB-231 group was much higher than that of the MCF-7 group (Fig. 2a), further showing the enhanced specificity accomplished by CCm coating. At 48 h after injection, all the mice were killed and the tumors and other major organs were collected for ex vivo UCL imaging. As shown in Fig. 2b, c, CCm_231_-UCNPs injected into MDA-MB-231 tumor-bearing mice displayed the highest rate of tumor accumulation. The amount of CCm_231_-UCNPs accumulated in the liver and spleen was much lower than that of UCNPs.

**Fig. 3:**
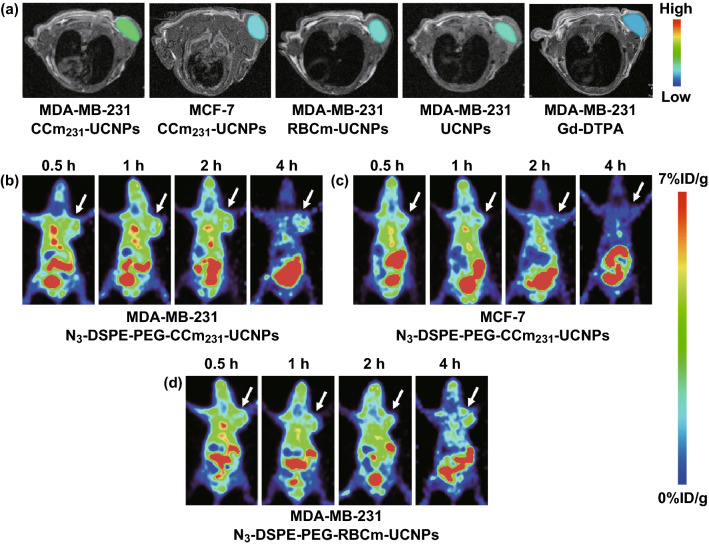
In vivo MR imaging. T1-weighted MR images acquired at 24 h after the injection of nanoparticles. **a** BALB/c nude tumor-bearing mice injected with Gd-DTPA or PBS containing CCm_231_-UCNPs, RBCm-UCNPs, UCNPs. The tumor sites are color-coded to better show the contrast-enhancing effects. In vivo PET imaging. Micro-PET static imaging was performed at 0.5, 1, 2, and 4 h after injection of Al[^18^F]F-L-NETA-DBCO. **b** MDA-MB-231 tumor-bearing mice injected with N_3_-DSPE-PEG-CCm_231_-UCNPs. **c** MCF-7 tumor-bearing mice injected with N_3_-DSPE-PEG-CCm_231_-UCNPs. **d** MDA-MB-231 tumor-bearing mice injected with N_3_-DSPE-PEG-RBCm-UCNPs

For in vivo MR imaging, the groups were set the same as those for UCL imaging. T1-weighted MR images were obtained at 24 h after the injection. The images of the Gd-DTPA group were acquired immediately after the injection. The group injected with CCm_231_-UCNPs showed slight enhancement in the tumor region compared to the groups injected with RBCm-UCNPs and UCNPs (Fig. 3a). Similarly, the tumor regions of CCm_231_-UCNPs injected into the MDA-MB-231 tumor groups were slightly enhancing than the CCm_231_-UCNPs injected into the MCF-7 groups (Fig. 3a).

For PET imaging, we used pre-targeting technology and click chemistry (Scheme S1). 1, 2-Distearoyl-sn-glycero-3-phosphoethanolamine-N-[azido (polyethylene glycol)-2000] (DSPE-PEG-N_3_) was inserted into CCm_231_ to obtain N_3_-PEG-DSPE-CCm_231_-UCNPs [32]. According to the UCL imaging, we chose to inject N_3_-DSPE-PEG-CCm_231_-UCNPs 24 h before the injection of ^18^F-labeled radioligands Al[^18^F]F-L-NETA-DBCO [33, 34]. The radioactive stability results are shown in Fig. S3. The cancer cells were injected into the upper limb of the mice to avoid the interference of the intestine and bladder on the imaging of the tumor area. Micro-PET static imaging was performed at 0.5, 1, 2, and 4 h after injection of Al[^18^F]F-L-NETA-DBCO. Images indicated that the N_3_-DSPE-PEG-CCm_231_-UCNPs group showed the highest uptake on tumor site at various times compared with other groups (Figs. 3b–d and S4). Also, the liver and spleen uptake were low. Injecting N_3_-DSPE-PEG-CCm_231_-UCNPs into MDA-MB-231 and MCF-7 tumor-bearing mice, respectively, the tumor site uptake of the MDA-MB-231 group was much higher than that of the MCF-7 group (Fig. 3b, c). The results were in accordance with that of UCL and MR imaging. Considering all these multimodality imaging results, we demonstrated that CCm_231_-UCNPs targeted to MDA-MB-231 tumors and were of greatly reduced immunogenicity.

### Biodistribution of CCm_231_-UCNPs

**Fig. 4:**
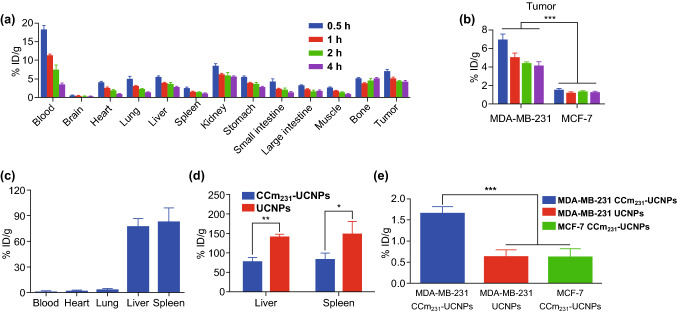
**a** Biodistribution of ^18^F in different organs and tumors in MDA-MB-231 tumor-bearing mice at different times after the injection. **b** Tumor uptake of ^18^F comparing in MDA-MB-231 and MCF-7 tumor-bearing mice. Biodistribution of Gd^3+^ in different organs, tissues, and tumors. **c** CCm_231_-UCNPs in the blood, heart, lung, liver, and spleen. **d** CCm_231_-UCNPs and UCNPs in the liver and spleen. **e** Tumor uptake of Gd^3+^ in the MDA-MB-231 CCm_231_-UCNPs group, the MDA-MB-231 UCNPs group, and the MCF-7 CCm_231_-UCNPs group. *, **, and *** indicate *P* < 0.05, *P* < 0.01, and *P* < 0.001, respectively. Data points represent mean ± SD (*n* = 4)

To quantitatively analyze the biodistribution of the probe, all the mice were killed, and blood samples, tumors, and major organs were collected for biological distribution by an automatic gamma counter. We injected N_3_-DSPE-PEG-CCm_231_-UCNPs 24 h in advance, and the biodistribution was performed at 0.5, 1, 2, and 4 h after the injection of Al[^18^F]F-L-NETA-DBCO. Comparing tumor uptake of ^18^F by the CCm_231_-UCNPs to MCF-7 model group (Table S2), CCm_231_-UCNPs to MDA-MB-231 model group (Table S3) showed significantly higher accumulation in the tumor (*P* < 0.001) (Fig. 4a, b); the results were in accord with that of imaging. Furthermore, the much higher tumor-to-blood ratios (T/B) and tumor-to-muscle ratios (T/M) of MDA-MB-231 model group were seen than those of MCF-7 model group (*P* < 0.05) (Table S2). It proved that CCm-coated nanoparticles had homologous-targeting ability. It could be inferred that the homologous-targeting ability of CCm-UCNPs can be widely applied to other types of tumors.

The detection of the ^18^F signal can only indirectly determine the biodistribution of nanoparticles, while directly detecting the distribution of Gd^3+^ in various tissues in the body must be achieved by inductively coupled plasma-atomic emission spectroscopy (ICP-AES). The results showed that the biodistribution of CCm_231_-UCNPs was mainly concentrated in the liver and spleen, and was rarely distributed in blood, heart, and lung (Fig. 4c and Table S4). The Gd^3+^ uptake in the liver measured by ICP-AES for UCNPs was 1.82-fold higher than CCm_231_-UCNPs (*P* < 0.001), and splenic uptake of Gd^3+^ for UCNPs was 1.79-fold greater than for CCm_231_-UCNPs (*P* < 0.05) (Fig. 4d). These results indicated that the uptake of CCm_231_-UCNPs by the liver and spleen was greatly reduced after coating with CCm, which proved their lowered immunogenicity. Tumor uptake of Gd^3+^ for CCm_231_-UCNPs was 2.62-fold higher than UCNPs (*P* < 0.001) (Fig. 4e). Also, the Gd^3+^ uptake in the tumor for CCm_231_-UCNPs to the MDA-MB-231 model group was 2.70-fold higher than that in CCm_231_-UCNPs to MCF-7 model group (*P* < 0.001) (Fig. 4e). There were significant differences in tumor uptake of Gd^3+^, showing that CCm_231_-UCNPs can homologously target tumors. The minimal uptake of UCNPs in the tumors may be attributed to the EPR effect. The difference between the MDA-MB-231 and MCF-7 model groups proved that CCm_231_-UCNPs could be used for the molecular classification of breast cancer in the future.

### In Vivo Toxicity Evaluation

**Fig. 5:**
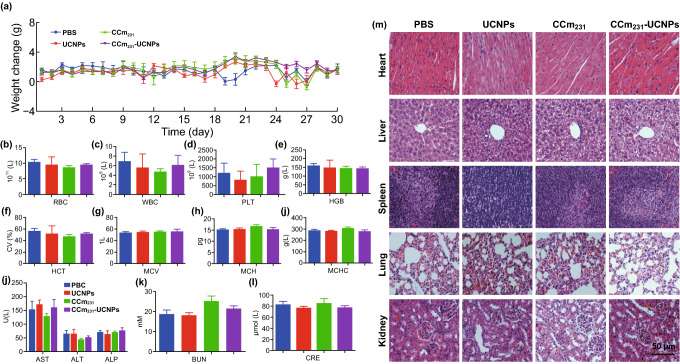
In vivo toxicity evaluation. **a** Mice body-weight-change curves over 30 days after i.v. injection with PBS or PBS containing UCNPs, CCm_231_, and CCm_231_-UCNPs. **b**–**i** Blood parameter data. Red blood cells (RBCs), white blood cells (WBCs), platelets (PLT), hemoglobin (HGB), hematocrit (HCT), mean corpuscular volume (MCV), mean corpuscular hemoglobin (MCH), and mean corpuscular hemoglobin concentration (MCHC). **j**–**l** Blood biochemistry data. Alkaline phosphatase (ALP), aspartate aminotransferase (AST), alanine transaminase (ALT), creatinine (CRE), and blood urea nitrogen (BUN). **m** H&E-stained slice images of major organs. The scale bars = 50 μm. The data points represent the mean ± SD (*n* = 6)

There is always a concern about potential toxicity and whether cancer cells or their membranes confer a cancer risk. For evaluating systematic toxicity, BALB/c nude mice received an injection of PBS, or PBS containing UCNPs, CCm_231_-vesicles, or CCm_231_-UCNPs. Neither death nor significant differences in body weight among the four groups were observed after 30 days (Fig. 5a). On the 30th day after the injection, all the mice were euthanized and their blood and major organs were collected for blood biochemistry, hematology tests, and histology analysis. We observed no significant differences between the treatment groups and control groups in the blood parameters and blood biochemistry indicators (Fig. 5b–l), and no significant organ damage on hematoxylin and eosin (H&E)-stained slices (Fig. 5m). Above all, the results suggested no obvious toxicity or carcinogenicity of CCm_231_-UCNPs in vivo.

## Conclusion

In conclusion, we designed a novel probe using MDA-MB-231 cancer cell membrane-mimic Gd^3+^-doped upconversion nanoparticles (CCm-UCNPs). In this probe, natural cell membranes isolated from cancer cells were coated into the UCNPs. The probe exhibited homologous-targeting and immune escaping abilities. Together with the upconversion luminescence of UCNPs, the paramagnetism of Gd^3+^, and click chemistry with surface modification to label ^18^F, CCm-UCNPs were used for ultra-sensitive in vivo UCL/MRI/PET multimodality precise imaging of TNBC, and differentiating MDA-MB-231 and MCF-7 tumor-bearing mice models in vivo.

Based on these results, this probe can also be modified for drug delivery, contributing to the treatment of TNBC. It may also be a potential method to achieve integration of diagnosis and treatment [35], as well as to monitor and evaluate therapeutic effects. In addition, apart from cancer cell membranes, various membrane functions, including chemotaxis of platelets to atherosclerotic plaque [25–27, 36], chemotaxis of leukocytes to inflammation [37], phagocytosis of macrophages [38], and cancer-targeting capabilities of stem cells [39], have the potential to be used for targeting different lesions. We believe this field has promising application prospects.

## Electronic supplementary material

Below is the link to the electronic supplementary material.

Supplementary material 1 (PDF 447 kb)
