# Supplementary material for: Ultra-sensitive Nanoprobe Modified with Tumor Cell Membrane for UCL/MRI/PET Multimodality Precise Imaging of Triple-Negative Breast Cancer
Source: Nanomicro Lett. 2020 Feb 22;12:62. doi: 10.1007/s40820-020-0396-4 (PMC7770711; doi:10.1007/s40820-020-0396-4)
Supplement: Supplementary file 1 — Supplementary material 1 (PDF 447 kb) [file 40820_2020_396_MOESM1_ESM.pdf]

Supporting Information for

Ultra-sensitive Nanoprobe Modified with Tumor Cell Membrane for UCL/MRI/PET Multimodality Precise Imaging of Triple-negative Breast Cancer

Hanyi Fang[1, 2], Mengting Li[1, 2], Qingyao Liu[1, 2], Yongkang Gai[1, 2], Lujie Yuan[1, 2], Sheng Wang[3], Xiao Zhang[1, 2], Min Ye[1, 2], Yongxue Zhang[1, 2], Mingyuan Gao[4], Yi Hou[5, *], Xiaoli Lan[1, 2, *]

[1]Department of Nuclear Medicine, Union Hospital, Tongji Medical College, Huazhong University of Science and Technology, Wuhan 430022, People's Republic of China

[2]Hubei Province Key Laboratory of Molecular Imaging, Wuhan 430022, People's Republic of China

[3]School of Pharmacy, Tongji Medical College, Huazhong University of Science and Technology, Wuhan 430030, People's Republic of China

[4]Center for Molecular Imaging and Nuclear Medicine, State Key Laboratory of Radiation Medicine and Protection, School for Radiological and Interdisciplinary Sciences (RAD-X), Soochow University, Suzhou, Jiangsu 215123, People's Republic of China

[5]Key Laboratory of Colloid, Interface and Chemical Thermodynamics, Institute of Chemistry, Chinese Academy of Sciences, Beijing 100190, People's Republic of China

*Corresponding authors. E-mail: xiaoli_lan@hust.edu.cn (Lan X.); houyi@iccas.ac.cn (Hou Y.)

S1 Supplementary Scheme

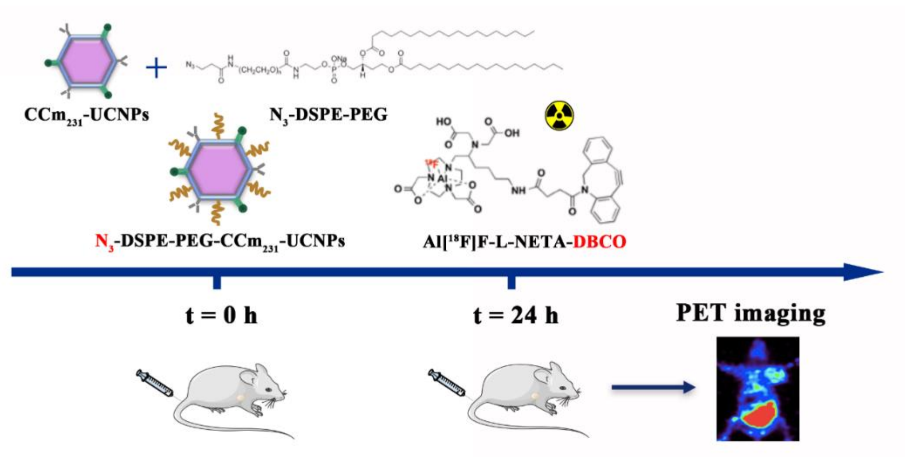

Scheme S1 First, 1, 2-distearoyl-sn-glycero-3-phosphoethanolamine-N-[azido (polyethylene glycol)-2000] (DSPE-PEG-N_3) was inserted into CCm_{231} to obtain N_3-PEG-DSPE-CCm_{231}-UCNPs and injected into tumor-bearing mice via the tail vein to achieve the pre-targeting. Then, 24 h later, L-NETA-DBCO were successfully radiolabeled with ^{18}F via Al-^{18}F chelation, and then ^{18}F-labeled aza-dibenzocyclooctyne (DBCO) radioligands (Al[^{18}F]F-L-NETA-DBCO) were injected into the tumor-bearing mice, and conjugated with N_3-PEG-DSPE-CCm_{231}-UCNPs by in vivo strain-promoted alkyne azide cycloaddition (SPAAC), which enables PET imaging.

S2 Supplementary Figures

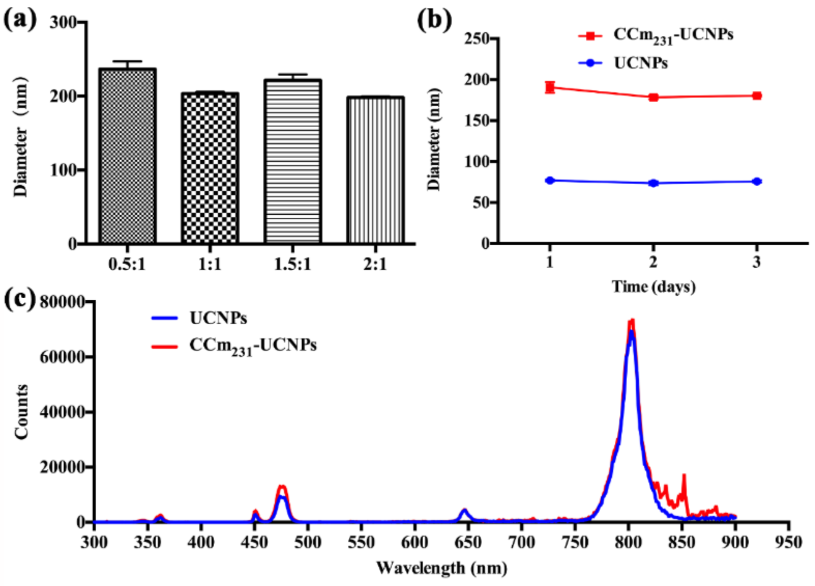

Fig. S1 Characterization of UCNPs and CCm_{231}-UCNPs. **a** Size of different proportions of CCm_{231} and UCNPs. **b** Stability of UCNPs and CCm_{231}-UCNPs. **c** Spectrum of UCNPs and CCm_{231}-UCNPs excited by 980 nm fluorescence

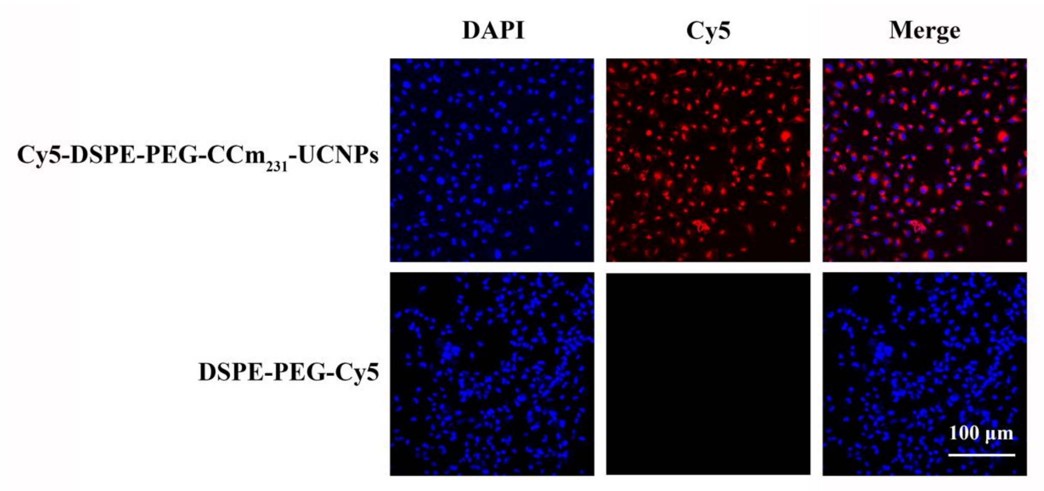

Fig. S2 The confocal laser scanning microscopy (CLSM) results of MDA-MB-231 cells uptake of Cy5-DSPE-PEG-CCm231-UCNPs and DSPE-PEG-Cy5. The scale bars is 100 um

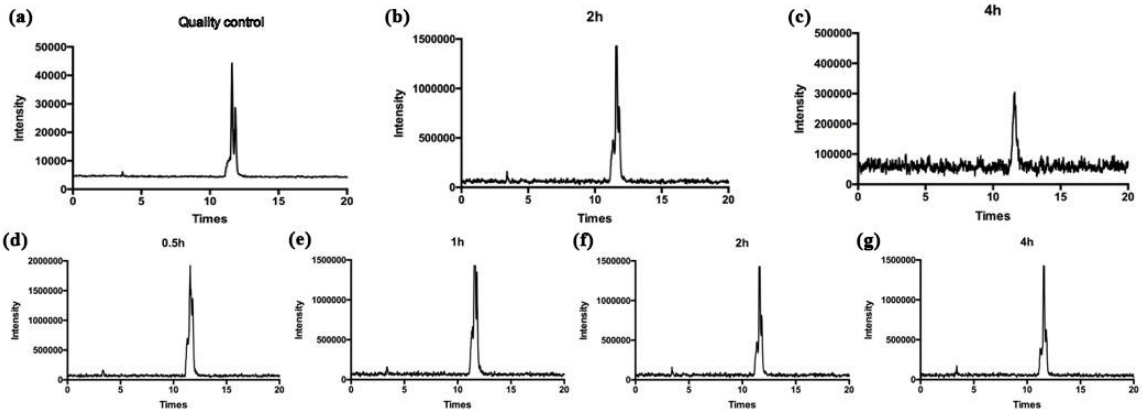

Fig. S3 Stability of the probe Al[^{18}F]F-L-NETA-DBCO. **a** Quality control of the probe Al[^{18}F]F-L-NETA-DBCO by HPLC. **b-d** In vivo stability of Al[^{18}F]F-L-NETA-DBCO by HPLC. **d-g** In vitro stability of Al[^{18}F]F-L-NETA-DBCO by HPLC

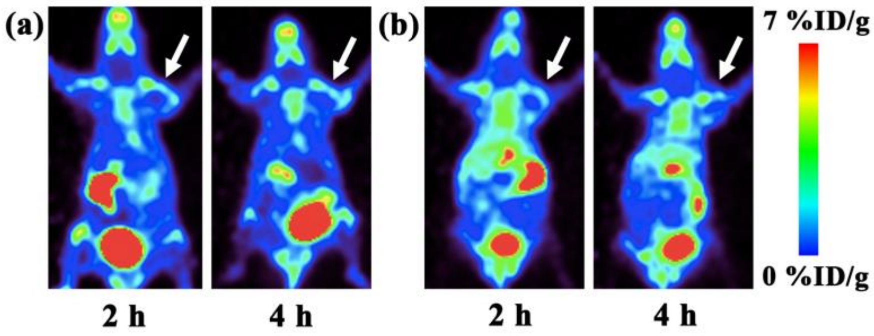

Fig. S4 a MDA-MB-231 tumor-bearing mice injected with Al[^{18}F]F-L-NETA-DBCO. **b** MDA-MB-231 tumor-bearing mice injected with DSPE-PEG-N_3. The white arrows indicate the sites of tumors

S3 Supplementary Tables

Table S1 The phenotype of different breast cancer cells

Cells	ER	PR	Her-2
MDA-MB-231	-	-	-
MCF-7	+	+	-
ZR-75-1	+	+	+
MDA-MB-453	-	-	+

Table S2 Biodistribution of ^{18}F labeled N_3-DSPE-PEG-CCm_{231}-UCNPs in different organs, tissues and tumors of MCF-7 tumor-bearing mice at different times after the injection. Data represent % ID/g, data points represent the mean ± SD (n=4). All data had been corrected for decay

**Tissues**	**0.5 h**	**1 h**	**2 h**	**4 h**
**Blood**	18.91 ± 1.86	13.04 ± 0.96	8.51 ± 1.47	2.60 ± 0.26
**Brain**	0.48 ± 0.11	0.31 ± 0.04	0.21 ± 0.05	0.09 ± 0.02
**Heart**	4.39 ± 0.55	2.58 ± 0.26	1.80 ± 0.24	0.68 ± 0.06
**Lung**	4.19 ± 1.25	2.86 ± 0.52	2.37 ± 0.31	1.08 ± 0.10
**Liver**	5.33 ± 0.37	3.94 ± 0.19	3.39 ± 0.37	2.15 ± 0.10
**Spleen**	2.05 ± 0.10	1.64 ± 0.08	1.33 ± 0.21	0.74 ± 0.03
**Kidney**	7.66 ± 0.73	5.62 ± 0.34	5.95 ± 0.48	4.80 ± 0.23
**Stomach**	2.41 ± 0.33	1.66 ± 0.07	1.18 ± 0.19	0.54 ± 0.04
**Small Intestine**	3.87 ± 0.77	2.97 ± 0.56	1.94 ± 0.31	0.62 ± 0.07
**Large Intestine**	2.92 ± 0.17	2.05 ± 0.28	1.50 ± 0.16	1.46 ± 0.32
**Muscle**	3.03 ± 1.31	1.93 ± 0.27	1.49 ± 0.12	2.23 ± 2.97
**Bone**	4.44 ± 0.53	3.47 ± 0.29	3.84 ± 0.28	8.82 ± 9.05
**Tumor**	1.48 ± 0.18	1.16 ± 0.10	1.28 ± 0.09	1.20 ± 0.14
**T/B**	0.08 ± 0.01	0.09 ± 0.01	0.15 ± 0.03	0.44 ± 0.01
**T/M**	0.53 ± 0.12	0.61 ± 0.12	0.87 ± 0.12	2.34 ± 0.10

Table S3 Biodistribution of ^{18}F labeled N_3-DSPE-PEG-CCm_{231}-UCNPs in different organs, tissues and tumors of MDA-MB-231 tumor-bearing mice at different times after the injection. Data represent % ID/g, data points represent the mean ± SD (n=4). All data had been corrected for decay

**Tissues**	**0.5 h**	**1 h**	**2 h**	**4 h**
**Blood**	18.12 ± 1.21	11.16 ± 0.42	7.30 ± 1.41	3.29 ± 0.61
**Brain**	0.41 ± 0.10	0.23 ± 0.02	0.18 ± 0.04	0.12 ± 0.03
**Heart**	3.92 ± 0.37	2.26 ± 0.47	1.74 ± 0.25	0.92 ± 0.15
**Lung**	4.84 ± 0.84	2.88 ± 0.28	2.15 ± 0.25	1.26 ± 0.18
**Liver**	5.37 ± 0.37	3.25 ± 1.35	3.48 ± 0.57	2.74 ± 0.20
**Spleen**	2.39 ± 0.33	1.46 ± 0.06	1.25 ± 0.17	0.88 ± 0.10
**Kidney**	8.29 ± 0.72	6.14 ± 0.29	5.76 ± 0.89	5.42 ± 0.31
**Stomach**	2.30 ± 0.25	1.32 ± 0.11	1.14 ± 0.27	0.62 ± 0.14
**Small Intestine**	4.08 ± 0.96	1.65 ± 0.97	2.35 ± 1.20	1.20 ± 0.21
**Large Intestine**	3.10 ± 0.11	2.01 ± 0.16	1.49 ± 0.36	1.57 ± 0.39
**Muscle**	2.45 ± 0.29	1.57 ± 0.21	1.21 ± 0.13	0.69 ± 0.16
**Bone**	4.94 ± 0.17	3.68 ± 0.20	4.35 ± 0.72	4.93 ± 0.40
**Tumor**	6.39 ± 1.12	4.97 ± 0.52	4.70 ± 0.90	4.05 ± 0.50
**T/B**	0.35 ± 0.04	0.44 ± 0.03	0.65 ± 0.09	1.28 ± 0.36
**T/M**	2.60 ± 0.28	3.19 ± 0.51	3.88 ± 0.53	6.01 ± 0.86

Table S4 Biodistribution of Gd^{3+} in different organs, tissues and tumors of MDA-MB-231 and MCF-7 tumor-bearing mice after the injection of CCm_{231}-UNCPs and UCNPs respectively. Data represent ‰ ID/g, data points represent the mean ± SD (n=4)

**Tumor models**	**MDA-MB-231 CCm_{231}-UCNPs**	**MDA-MB-231 UCNPs**	**MCF-7 CCm_{231}-UCNPs**
**Blood**	0.30 ± 0.07	\	\
**Liver**	77.04 ± 9.85	139.86 ± 8.22	\
**Spleen**	82.42 ± 16.97	147.69 ± 33.52	\
**Tumor**	1.65 ± 0.16	0.63 ± 0.17	0.61 ± 0.21
